# Deletion of inositol hexakisphosphate kinase 1 (IP6K1) reduces cell migration and invasion, conferring protection from aerodigestive tract carcinoma in mice

**DOI:** 10.1016/j.cellsig.2016.04.011

**Published:** 2016-08

**Authors:** Rathan S. Jadav, Dharmika Kumar, Natasha Buwa, Shubhra Ganguli, Sitalakshmi R. Thampatty, Nagaraj Balasubramanian, Rashna Bhandari

**Affiliations:** a Laboratory of Cell Signalling, Centre for DNA Fingerprinting and Diagnostics, Hyderabad 500001, India; b Graduate Studies, Manipal University, Manipal, India; c Department of Biology, Indian Institute of Science Education and Research, Pune 411008, India

**Keywords:** 4NQO, 4-nitroquinoline-1-oxide, 5-IP_7_, 5-diphosphoinositol 1, 2, 3, 4, 6-pentakisphosphate, BP, biological process, CC, cellular component, DMEM, Dulbecco's modified Eagle's medium, ECM, extracellular matrix, FAK, focal adhesion kinase, FBS, fetal bovine serum, IP_6_, inositol hexakisphosphate, IP6K, inositol hexakisphosphate kinase, GO, gene ontology, H&E, hematoxylin and eosin, MEFs, mouse embryonic fibroblasts, MF, molecular function, NT, non-targeting, RT-qPCR, reverse transcription quantitative polymerase chain reaction, Inositol hexakisphosphate kinase, Inositol pyrophosphate, Gene expression, Cell migration, Cell invasion, Aerodigestive tract carcinoma

## Abstract

Inositol hexakisphosphate kinases (IP6Ks), a family of enzymes found in all eukaryotes, are responsible for the synthesis of 5-diphosphoinositol pentakisphosphate (5-IP_7_) from inositol hexakisphosphate (IP_6_). Three isoforms of IP6Ks are found in mammals, and gene deletions of each isoform lead to diverse, non-overlapping phenotypes in mice. Previous studies show a facilitatory role for IP6K2 in cell migration and invasion, properties that are essential for the early stages of tumorigenesis. However, IP6K2 also has an essential role in cancer cell apoptosis, and mice lacking this protein are more susceptible to the development of aerodigestive tract carcinoma upon treatment with the oral carcinogen 4-nitroquinoline-1-oxide (4NQO). Not much is known about the functions of the equally abundant and ubiquitously expressed IP6K1 isoform in cell migration, invasion and cancer progression. We conducted a gene expression analysis on mouse embryonic fibroblasts (MEFs) lacking IP6K1, revealing a role for this protein in cell receptor-extracellular matrix interactions that regulate actin cytoskeleton dynamics. Consequently, cells lacking IP6K1 manifest defects in adhesion-dependent signaling, evident by lower FAK and Paxillin activation, leading to reduced cell spreading and migration. Expression of active, but not inactive IP6K1 reverses migration defects in IP6K1 knockout MEFs, suggesting that 5-IP_7_ synthesis by IP6K1 promotes cell locomotion. Actin cytoskeleton remodeling and cell migration support the ability of cancer cells to achieve their complete oncogenic potential. Cancer cells with lower IP6K1 levels display reduced migration, invasion, and anchorage-independent growth. When fed an oral carcinogen, mice lacking IP6K1 show reduced progression from epithelial dysplasia to invasive carcinoma. Thus, our data reveal that like IP6K2, IP6K1 is also involved in early cytoskeleton remodeling events during cancer progression. However, unlike IP6K2, IP6K1 is essential for 4NQO-induced invasive carcinoma. Our study therefore uncovers similarities and differences in the roles of IP6K1 and IP6K2 in cancer progression, and we propose that an isoform-specific IP6K1 inhibitor may provide a novel route to suppress carcinogenesis.

## Highlights

• Gene-expression analysis of *Ip6k1* knockout MEFs reveals down-regulation of cell surface-extracellular matrix signaling.
• *Ip6k1* knockout MEFs show reduced adhesion-dependent signaling, cell spreading and migration.
• Cancer cells deficient in IP6K1 display reduced migration, invasion and anchorage independent growth.
• Mice lacking IP6K1 are resistant to progression from epithelial dysplasia to invasive carcinoma.

## Graphical abstract

**Figure:**
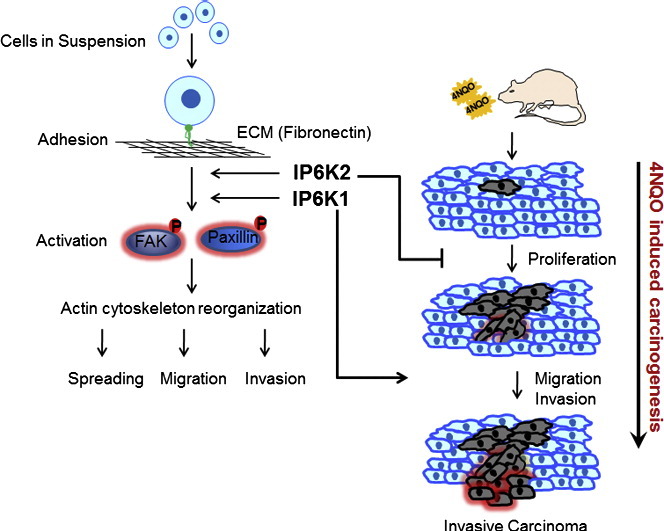

## 1 Introduction

Inositol pyrophosphates are a class of high-energy signaling molecules characterized by the presence of pyrophosphate and monophosphate substitutions on the inositol ring [1], [2], [3], [4]. They participate in many cellular functions by binding specific proteins or by transferring their β phosphate to prephosphorylated serine residues to bring about serine pyrophosphorylation [3], [4], [5], [6]. The most abundant inositol pyrophosphate in mammals, 5-diphosphoinositol pentakisphosphate (5-PP-IP_5_ or 5-IP_7_) is synthesized from inositol hexakisphosphate (IP_6_) by inositol hexakisphosphate kinases (IP6Ks) [7], [8]. IP6Ks can also synthesize 5-PP-IP_4_ from IP_5_ [8], [9]. IP6Ks are found in all eukaryotes, with lower organisms such as yeast, slime mold and *Drosophila* possessing a single IP6K, and mammals possessing three IP6K isoforms, IP6K1, 2 and 3, encoded by distinct genes [10]. Amongst these, IP6K1 and IP6K2 are expressed in several tissues [7], whereas IP6K3 shows high expression only in the cerebellum [9].

IP6K1 promotes insulin release from pancreatic β cells [11], and participates in maintaining genome integrity via homologous recombination mediated DNA repair [12] and nucleotide excision repair [13]. Mice lacking IP6K1 display reduced serum insulin [14], male infertility [14], decreased susceptibility to a thrombotic challenge [15], enhanced Akt signaling [16], and reduced social behavior [17]. IP6K2 has been shown to promote tumor cell growth and migration by antagonizing liver kinase B1 [18]. Conversely, IP6K2 also enhances p53-mediated apoptosis in cancer cells so that the loss of IP6K2 results in reduced apoptosis [19], [20], and IP6K2 knockout mice are more susceptible to 4-nitroquinoline-1-oxide (4NQO) induced aerodigestive tract carcinoma [21]. Recently, mice lacking IP6K3 were shown to display defects in motor function due to altered cytoskeletal architecture in cerebellar Purkinje cells [22].

Interaction of cells with the extracellular matrix triggers adhesion-dependent signaling pathways that play an important role in the regulation of cell growth, survival, cell migration and invasion - processes that are crucial in the pathophysiology of cancer [23]. Upon exposure to a carcinogen, epithelial cells display hyperproliferation and undergo structural and biochemical changes that aid in their migration and invasion into the underlying basement membrane [24], [25]. Eventually, these tumor cells can invade blood and lymphatic vessels to metastasize to other tissues.

To explore the biological functions of IP6K1, we conducted a microarray-based gene expression analysis on mouse embryonic fibroblasts (MEFs) derived from *Ip6k1*^−/−^ mice. Interestingly, cells lacking IP6K1 showed down-regulated expression of several genes involved in mediating cell–matrix interaction and downstream signaling. Consequently, cells with reduced IP6K1 expression displayed diminished migration and invasion properties. Contrary to observations made in *Ip6k2* knockout mice, deletion of *Ip6k1* led to reduced development of invasive epithelial carcinoma upon chronic exposure of the aerodigestive tract to 4NQO. Therefore, our data shows that IP6K1 expression is required for cancer cells to achieve their complete oncogenic potential.

## 2 Materials and methods

### 2.1 Cell lines

All cell lines were grown at 37 °C in a humidified incubator with 5% CO_2_. *Ip6k1*^+/+^ and *Ip6k1*^*−*/*−*^ MEFs [14] and *Ip6k1*^*−*/*−*^ MEFs expressing kinase active or inactive variants of IP6K1 [12] were cultured in Dulbecco's modified Eagle's medium (DMEM, Life Technologies) supplemented with 10% fetal bovine serum (FBS, Life Technologies), 1 mM l-Glutamine (Life Technologies), 100 U/mL penicillin, and 100 μg/mL streptomycin (Life Technologies). HeLa and HCT116 cell lines were used for stable knockdown of IP6K1 expression. Lentiviral vectors (pLKO.1) encoding either a non-targeting shRNA (SHC016, Sigma-Aldrich) or two specific sequences of shRNA directed against human *IP6K1* (TRC0000013508, designated sh*IP6K1–1* and TRC0000196808, designated sh*IP6K1–4*, Sigma-Aldrich), were co-transfected with VSV-G and psPAX2 (a gift from Didier Trono, Addgene plasmid #12260) plasmids into the HEK293T packaging cell line, using polyethylenimine reagent (Polysciences Inc.) and incubated at 37 °C with 5% CO_2_ to generate lentiviral particles. After 48 h, viral particles were harvested from the culture supernatant by filtering through a 0.45 μm syringe filter unit. Viral particles harboring either non-targeting control or *IP6K1* directed shRNA were used to infect HeLa or HCT116 cell lines following treatment with polybrene (8 μg/mL, Sigma-Aldrich) for 2 h. After 48 h, transduced cells were selected with 2 μg/mL puromycin (Sigma-Aldrich) by changing the medium twice a week. Once cells reached optimal growth, polyclonal populations were maintained in complete DMEM supplemented with 1 μg/mL puromycin (Sigma-Aldrich). Knockdown was confirmed by immunoblot analysis with an IP6K1 specific antibody.

### 2.2 Mice

All animal experiments were conducted as per guidelines provided by the Committee for the Purpose of Control and Supervision of Experiments on Animals, Ministry of Environment, Forest, and Climate Change, Government of India, and these experiments were approved by the Institutional Animal Ethics Committee (Protocol numbers PCD/CDFD/02-version 2 and PCD/CDFD/08). Mice used for this study were housed in the Centre for DNA Fingerprinting and Diagnostics animal facility located within the premises of Vimta Labs, Hyderbad. *Ip6k1*^+/*−*^ heterozygous mice were bred to generate age and sex matched *Ip6k1*^+/+^ and *Ip6k1*^*−*/*−*^ littermates for experiments. *Foxn1*^*nu*^ mice were generated by breeding homozygous males with heterozygous females.

### 2.3 Reagents and antibodies

Primary antibodies used for immunoblot analysis were obtained from the following sources: Rabbit anti-IP6K1 (HPA040825, Sigma-Aldrich), goat anti-IP6K1 (sc-10419, Santa Cruz Biotechnology), rabbit anti-phosphoFAK (Tyr397) (3283, Cell Signaling Technology), rabbit anti-FAK (3285, Cell Signaling Technology), rabbit anti-phosphoPaxillin (Tyr118) (2541, Cell Signaling Technology), mouse anti-Paxillin (610051, BD Biosciences), mouse anti-actin (ab3280, Abcam), mouse anti-GAPDH (G8795, Sigma-Aldrich), mouse anti-E Cadherin (14472S, Cell Signaling Technology), and rabbit anti-vimentin (ab92547, Abcam). Reagents used for cell spreading: fibronectin (F2006, Sigma-Aldrich), methyl cellulose (Sigma-Aldrich) and fluorophore conjugated phalloidin (Molecular Probes Inc.). Propylene glycol (151957, MP Biomedicals) was used to dissolve the oral carcinogen 4-Nitroquinoline-1-Oxide (4NQO, Sigma-Aldrich). All other reagents, unless otherwise stated, were obtained from Sigma-Aldrich.

### 2.4 Gene expression microarray

MEFs derived from two *Ip6k1*^+/+^ and *Ip6k1*^−/−^ embryos each were immortalised by the expression of SV40 large T antigen [14] and stored in liquid nitrogen. Thawed cells were passaged twice, harvested by trypsinization, and their genotype was re-confirmed by PCR as described in [14]. 10^7^ cells were resuspended in 100 μL PBS and 5 volumes of RNAlater (Sigma-Aldrich). Total RNA was extracted using the RNeasy Mini Kit (Qiagen). The samples were labeled using the Agilent Quick-Amp labeling Kit. 500 ng of each sample was incubated with reverse transcription mix at 40 °C and converted to double stranded cDNA primed by oligonucleotide dT with a T7 polymerase promoter. cRNA was generated by in vitro transcription of the double stranded cDNA at 40 °C, and the dye Cy3 CTP (Agilent) was incorporated during this step. 600 ng of Cy3 labeled RNA samples were fragmented and hybridized using the Gene Expression Hybridization kit (Agilent). Hybridization onto the Agilent-014868 Whole Mouse Genome Microarray 4x44K (G4122F) was carried out in Agilent's Surehyb Chambers at 65 °C for 16 h. The hybridized slides were washed using Gene Expression wash buffers (Agilent), and scanned using the Agilent Microarray Scanner (G2505C) at 5 μm resolution. Data was extracted using Feature Extraction software and normalized using GeneSpring GX (75th percentile shift method). Data are presented as the average fold change in gene expression in the two *Ip6k1*^*−*/*−*^ MEF lines compared with the average expression for that gene in two *Ip6k1*^+/+^ MEF lines.

Gene expression quantification was carried out by reverse transcription quantitative real time PCR (RT-qPCR). Briefly, total cellular RNA was isolated from single cell derived *Ip6k1*^+/+^ and *Ip6k1*^−/−^ MEFs using TRIzol reagent (Invitrogen) followed by RNeasy Mini Kit (Qiagen). 2 μg of total RNA was used for first strand cDNA synthesis by reverse transcription using SuperScript Reverse Transcriptase III (Invitrogen) with oligonucleotide dT primers. qPCR was performed using gene specific primers on the ABI 7500 Real-Time PCR System (Applied Biosystems) with MESA GREEN qPCR MasterMix Plus for SYBR® Assay Low ROX (Eurogentec) for detection. Each sample was run in duplicate. The primer sequences are listed in Supplemental Table S1. The difference in gene expression was calculated using the fold change (ΔΔC_t_ method) [26]. ΔC_t_ is the C_t_ value for the gene of interest normalized to the C_t_ value of the respective GAPDH control in both *Ip6k1*^+/+^ and *Ip6k1*^−/−^ MEFs. ΔΔC_t_ values were calculated as a relative change in ΔC_t_ of the target gene in *Ip6k1*^−/−^ with respect to *Ip6k1*^+/+^ MEFs. Fold changes were expressed as 2^− ΔΔCt^ for up-regulated genes and the negative reciprocal of the fold change for down-regulated genes (where 2^− ΔΔCt^ < 1).

### 2.5 Cell adhesion

Cell adhesion assays were performed as described previously [27] with slight modifications. Briefly, 5 × 10^4^ cells were seeded per well into fibronectin (2 μg/mL) coated 24 well plates. Cells were allowed to adhere for the indicated time. At each time point, unadhered cells were washed away with media and fresh medium was added to the wells. After 5 h, wells were washed with PBS and stained with 0.2% crystal violet (in 2% ethanol) for 10 min. Wells were thoroughly washed with PBS and cells were lysed by shaking in 1% SDS. Absorbance was monitored at 595 nm using an EnSpire multimode plate reader (PerkinElmer).

### 2.6 Cell spreading

Cell spreading assays were done as described previously [28]. Briefly, cells were cultured in complete medium (10% FBS) or subjected to serum starvation (0.2% FBS for 12–14 h), trypsinized, counted using a hemocytometer, and held in suspension in the same medium containing 1% methylcellulose at 37 °C and 5% CO_2_. After 90 min, cells were washed twice with medium to remove methylcellulose. Cell suspension equivalent to 10^5^ cells was plated onto each fibronectin (2 μg/mL) coated coverslip and allowed to spread for 15 min or 24 h in serum depleted (0.2% FBS) or complete medium. At each time point, the coverslips were fixed with 3.5–4% paraformaldehyde for 20 min, followed by 3 washes with PBS. Fixed cells were stained with fluorophore-conjugated phalloidin (Alexa Fluor 488 or rhodamine) for 45 min, followed by DAPI staining for 2 min. Cover slips were mounted onto glass slides using Fluoromount G (Southern Biotech) or Vectashield (Vector Labs), and imaged using an LSM 710 laser confocal-anisotropy microscope (Zeiss, Zen acquisition software, 40 × 1.3 N.A. objective) for 15 min spreading, or LSM 510 laser confocal microscope (Zeiss, LSM acquisition software, 63 × 1.4 N.A. objective) for 24 h spreading. To measure cell spread area, images captured at identical zoom settings were analyzed using the Image J software (NIH) as follows: threshold values were set to define the cell edge and a mask was then created for each cell to get the total cell area (with arbitrary units) within the mask. For 24 h spreading, the exact spread area was calculated based on pixel dimensions during image acquisition.

### 2.7 Western blot analysis

Cells were seeded in six well plates. After attaining optimal growth, cell lysates were prepared by scraping cells in 1 × Laemmli buffer and samples were processed by standard Western blot techniques. To detect FAK and Paxillin activation, 2 × 10^6^ cells (6 × 10^5^ cells per time point) were held in suspension in complete medium containing 1% methylcellulose for 90 min (Susp), and replated on fibronectin (2 μg/mL) coated surfaces for 20 min (+ FN) or for 4 h (SA - stably adherent). Cells at each time point were lysed in 1 × Laemmli buffer and subjected to immunoblotting. Membranes were probed with specific antibodies and detected using the ECL detection system (GE Healthcare). Chemiluminiscence was detected using the LAS4000 (GE Healthcare) or FlourChem E (Protein Simple) documentation system. Densitometry analysis of bands was done using ImageJ documentation software (Fiji) or the multiplex band analysis tool in AlphaView software (Protein Simple).

### 2.8 Cell proliferation assay

Cells were seeded in 24 well plates at 10% confluence in triplicates and allowed to grow for different lengths of time. Spent medium was replaced with fresh medium every 48 h. Cell viability was analyzed by the MTT assay at each time point [12]. Absorbance values at 570 nm were measured using the EnSpire multimode plate reader (PerkinElmer). Doubling time was determined by conducting linear regression analysis using GraphPad Prism 5.

### 2.9 Transwell migration and invasion assay

Transwell migration and invasion assays were conducted as described previously [18]. Briefly, transwell inserts (24 well, 8 μm pore size, Costar, Corning) with or without pre-coated matrigel (BioCoat Matrigel invasion chamber, 24 well, 8 μm pore size, Corning) were used to conduct the migration and invasion assays respectively. 5 × 10^4^ (for migration assay) or 1 × 10^5^ (for invasion assay) cells were suspended in 200 μL of DMEM medium containing 1% FBS and added to the upper chambers while the lower chambers were filled with 700 μL of complete DMEM containing 10% FBS to serve as the chemotactic agent. Cells were incubated at 37 °C with 5% CO_2_ for 18 h (MEFs) and 24 h (HeLa and HCT116 cells) to allow migration and invasion. At the indicated time, cells on the upper surface of the filters were removed using cotton swabs. Cells that migrated to the lower surface of the filter were rinsed with PBS twice, fixed with 4% paraformaldehyde for 15 min, and stained with DAPI. Images were acquired using an epifluorescence inverted microscope (Olympus lX51, Image-Pro AMS 6.0 acquisition software, 20 × 0.45 N.A. objective). The number of cells migrated to the lower surface was quantified by counting the total number of DAPI positive nuclei in at least 10 random fields. For the invasion assay, the total number of cells invaded was normalized to non-targeting control cells and expressed as percentage invasion.

### 2.10 Scratch wound healing assay

Scratch wound healing assay was performed as described previously [18].Cells were seeded in 6 well plates to attain confluence. On the next day, a scratch was made on the confluent monolayer culture using a 200 μL pipette tip. Wound closure was monitored during the indicated time points. Images were acquired using a Zeiss phase contrast inverted microscope (ProgRes CapturePro v2.8 acquisition software, 5 × 0.12 N.A. objective). The area of wound closure was analyzed using ImageJ software, and the data was plotted as total area covered in square μm.

### 2.11 Analysis of cellular inositol polyphosphates

Soluble inositol polyphosphate levels were analyzed as previously described [29]. 2.5 × 10^5^ cells cultured as described in Section 2.1 were labeled with 60 μCi [2 −^3^H] myo-inositol (MP Biomedicals, specific activity 23.75 Ci/mmol) for 4 days and subjected to extraction of soluble inositol phosphates [29]. The lipid inositol fraction was extracted as described earlier [14] and counted using a liquid scintillation analyzer (Tri-Carb 2910 TR, Perkin Elmer). Soluble inositol phosphates were resolved by HPLC on a Partisphere SAX column (4.6 mm × 125 mm, Whatman) using a gradient of Buffer A (1 mM EDTA) and Buffer B [1mM EDTA and 1.3 M (NH_4_)_2_HPO_4_ (pH 3.8)] as follows: 0–5 min, 0%B; 5–10 min, 0–20%B; 10–70 min, 20–100%B; 70–80 min, 100%B. 1 mL fractions containing soluble inositol phosphates were counted, normalized to the total lipid count, and plotted using GraphPad Prism 5.

### 2.12 Anchorage-independent cell growth

Anchorage independent cell growth was assessed as previously described [30]. Briefly, 16,000 cells were suspended in 0.3% top agar and plated in duplicates in 6 well plates containing a 0.5% base agar layer. Plates were incubated at 37 °C and 5% CO_2_ for 28 days. After every 2 days, 0.5 mL medium was applied to the top agar to prevent it from drying out. After 28 days, colonies were stained with 0.005% crystal violet (in 2% ethanol), imaged using an AlphaImager (Alpha Innotech), and the number of colonies was determined using ImageJ software. Images of stained colonies were acquired using a Zeiss phase contrast inverted microscope (ProgRes CapturePro v2.8 acquisition software, 5 × 0.12 N.A. objective). Area of the colonies was determined using ImageJ software.

### 2.13 In vivo xenograft model

In vivo xenograft studies were performed as previously described [18]. Approximately 2 × 10^6^ HCT116 cells stably expressing NT or shRNA against *IP6K1* were injected subcutaneously into either flank of 6 week old homozygous *Foxn1*^*nu*^ athymic nude mice (n = 8 mice). Mice were euthanized 4 weeks after injection and tumors were surgically excised and weighed.

### 2.14 Carcinogenesis study in Ip6k1 knockout mice

Carcinogenesis studies were conducted as described previously for *Ip6k2*^*−*/−^ mice [21]. Briefly, the carcinogen, 4NQO stock solution (5 mg/mL) was prepared fresh in propylene glycol every week. Four week old mice [11 *Ip6k1*^+/+^ (5 male, 6 female) and 9 *Ip6k1*^*−*/*−*^ (4 male, 5 female)] were exposed to 4NQO (100 μg/mL) in their drinking water. Mice were allowed free access to drinking water containing carcinogen, and the water was changed every week. After 24 weeks, mice were euthanized by CO_2_ inhalation and a complete necropsy was performed. Tissues from the aerodigestive tract (tongue, esophagus, stomach and duodenum) were fixed in formalin and paraffin embedded sections were stained with hematoxylin and eosin (H&E) to examine the lesions by light microscopy. Mice showing any characteristics of weight loss or dehydration before 24 weeks were euthanized and examined for lesions. Images were acquired using a bright field light microscope (Nikon ECLIPSE N*i*, NIS Elements acquisition software, 20 × 0.5 N.A. objective). During pathological examination, the lesions observed in the various tissues were categorized into hyperplasia, dysplasia and invasive carcinoma. Hyperplasia was defined as an increase in the layers or thickening of the epithelium with hyperkeratinization. Dysplasia was defined as loss of epithelial cell polarity, nuclear pleomorphism and abnormal mitoses confined to the epithelium. Invasive carcinoma was defined as invasion of dysplastic cells or lesions into subepithelial tissues such as submucosa and muscle.

### 2.15 Data analysis

Statistical analysis was performed using GraphPad Prism 5. Data are presented as mean ± SEM. The difference between two groups was analyzed using either a two-tailed Student's *t*-test or a nonparametric two-tailed Mann-Whitney test, as appropriate. The differences between multiple groups were analyzed by one way ANOVA, using Tukey's multiple comparison test. P ≤ 0.05 was considered as statistically significant. The cell numbers used to obtain quantitative data (n) and the number of independent experiments performed is indicated in the respective figure legends.

## 3 Results

### 3.1 Loss of IP6K1 leads to dysregulation of genes involved in cell surface-extracellular matrix signaling

**Fig. 1:**
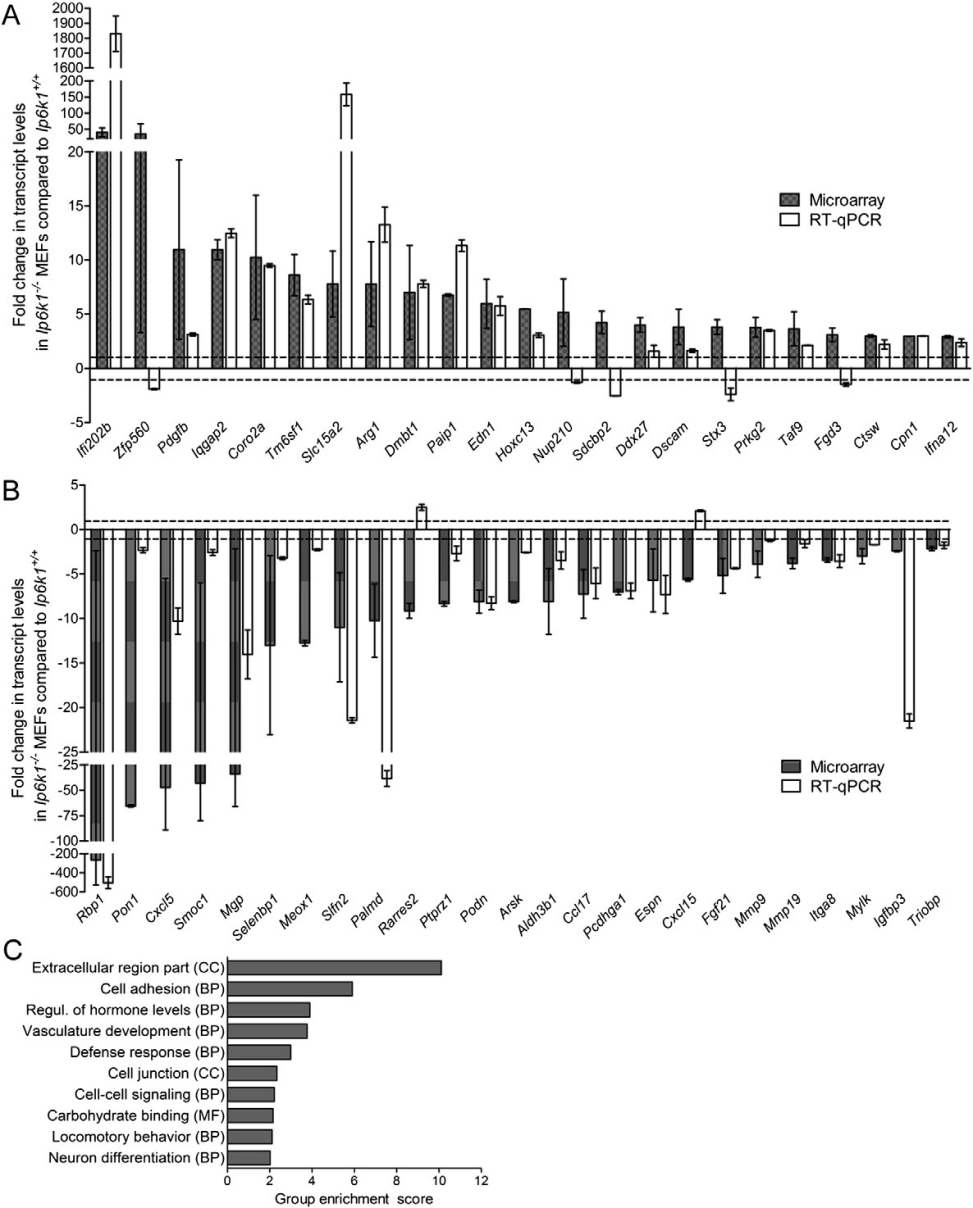
Deletion of *Ip6k1* in mice alters gene expression. RT-qPCR analysis of 23 up-regulated (A) and 25 down-regulated (B) genes each, to validate the gene expression microarray conducted on *Ip6k1*^+/+^ and *Ip6k1*^*−*/*−*^ MEFs. Values indicate the fold change in transcript levels in *Ip6k1*^*−*/*−*^ MEFs normalized to *Ip6k1*^+/+^ MEFs. The microarray data is the average fold change in gene expression in two independent *Ip6k1*^*−*/*−*^ MEF lines compared with the average expression for that gene in two independent *Ip6k1*^+/+^ MEF lines. RT-qPCR data (mean ± range from two independent experiments) is the fold change in gene expression in a single-cell derived *Ip6k1*^*−*/*−*^ MEF line compared with a single-cell derived *Ip6k1*^+/+^ MEF line. These cell lines were used for further studies. The dashed lines in the graphs mark the range from − 1 to + 1, the region of no change in gene expression. (C) The list of down-regulated genes in *Ip6k1*^*−*/*−*^ MEFs was subjected to Functional Annotation Clustering of Gene Ontology (GO) terms using the DAVID tool. The graph depicts clusters of Cellular Component (CC), Biological Process (BP) and Molecular Function (MF) GO terms with a group enrichment score ≥ 2 (P ≤ 0.01).

To uncover novel biological functions of IP6K1, we performed a gene expression microarray analysis on mouse embryonic fibroblast (MEF) lines derived from two *Ip6k1*^+/+^ and *Ip6k1*^−/−^ embryos each [14]. Applying a cutoff of 2-fold difference in expression in *Ip6k1*^−/−^ compared with *Ip6k1*^+/+^ MEFs, we observed 374 up-regulated and 888 down-regulated genes (Supplemental Table S2). To validate the data from the microarray, we selected 23 up- and 25 down-regulated genes spanning a wide range of fold changes in gene expression, and conducted RT-PCR analysis to compare transcript levels in *Ip6k1*^+/+^ and *Ip6k1*^−/−^ MEFs. Amongst the 48 genes tested 41 transcripts showed the same trend by RT-PCR and microarray (Fig. 1A–B), implying that the majority of dysregulated genes identified by microarray analysis are likely to display differential transcript levels in *Ip6k1*^−/−^ compared with *Ip6k1*^+/+^ MEFs.

The lists of up- and down-regulated genes were subjected to Functional Annotation Clustering using the DAVID tool [31] (Supplemental Table S2). We found significant enrichment of Gene Ontology (GO) term clusters with an enrichment score larger than 2 (P-value ≤ 0.01) only in the down-regulated gene list (Fig. 1C). In this gene list, enriched cellular component (CC) GO terms include proteins located in the extracellular region and cell junctions, and enriched biological process (BP) GO terms include cell adhesion, cell-cell signaling and locomotory behavior. The enrichment of these GO terms suggests that processes involving cell receptor and extracellular matrix interactions such as cell adhesion, spreading and migration may be deregulated in the absence of IP6K1.

### 3.2 IP6K1 is required for adhesion dependent cell spreading

**Fig. 2:**
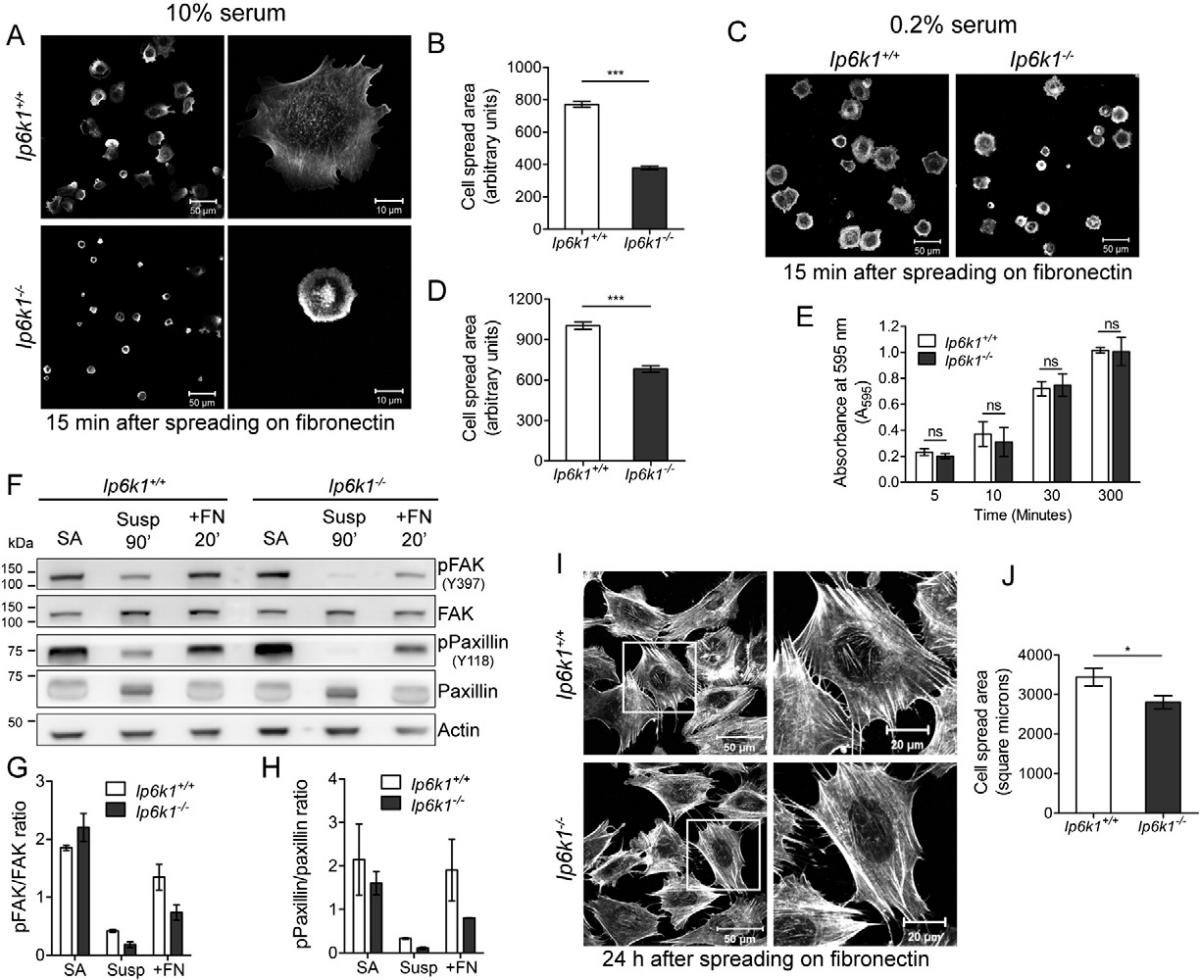
IP6K1 regulates cell spreading on fibronectin. (A) Spreading of *Ip6k1*^+/+^ and *Ip6k1*^*−*/*−*^ MEFs 15 min after plating on fibronectin in the presence of 10% serum, visualized by staining with Alexa Fluor 488-conjugated phalloidin. Scale bars represent 50 μm (left panel) and 10 μm (right panel). (B) Quantification of (A); the bar graph shows the cell spread area after 15 min in MEFs of the indicated genotypes. Data represents mean ± SEM (n = 438 and 474 cells respectively for *Ip6k1*^+/+^ and *Ip6k1*^*−*/*−*^ MEFs), compiled from four independent experiments and was analyzed using the non-parametric two-tailed Mann-Whitney test. (C) Spreading of MEFs 15 min after plating on fibronection in the presence of 0.2% serum, visualized as in (A). Scale bars represent 50 μm. (D) Quantification of (C); the bar graph shows the cell spread area after 15 min in MEFs of the indicated genotypes. Data represents mean ± SEM (n = 216 and 230 cells respectively for *Ip6k1*^+/+^ and *Ip6k1*^*−*/*−*^ MEFs), compiled from three independent experiments and was analyzed as in (B). (E) Adhesion of MEFs to a fibronectin-coated surface was monitored by crystal violet staining of adhered cells after washing off the unattached cells. The bar graph shows the crystal violet absorbance values at the indicated time points. Data are mean ± SEM from three independent experiments, analyzed by a two tailed unpaired Student's *t*-test. (F) Immunoblot analysis of FAK and Paxillin activation in MEFs conducted as described in the Methods section. Membranes were probed with the indicated antibodies and actin was used as the loading control. (G, H) Quantification of pFAK (G) and pPaxillin (H) levels from panel F. Data are mean ± range from two independent experiments. (I) Representative images showing cell morphology 24 h after spreading on fibronectin visualized by staining with rhodamine-conjugated phalloidin. Scale bars represent 50 μm (left panel) and 20 μm (right panel showing an enlarged image from the same field). (J) Quantification of (I); bar graph shows the cell spread area 24 h after plating. Data represents mean ± SEM (n = 20 and 22 cells respectively for *Ip6k1*^+/+^ and *Ip6k1*^*−*/*−*^ MEFs) compiled from two independent experiments and was analyzed using the non-parametric two-tailed Mann-Whitney test. ***P ≤ 0.001; *P ≤ 0.05; ns, not significant, P > 0.05.

Integrins are transmembrane heterodimeric receptors that engage with the extracellular matrix (ECM) to activate intracellular signaling at focal adhesions, leading to actin cytoskeleton remodeling [32]. There is extensive cross-talk of integrin-mediated signaling with growth factor- mediated signaling pathways, so that on loss of adhesion several growth factor dependent signaling pathways are also down-regulated [33]. Subsequent readhesion of cells restores signaling to drive cytoskeletal remodeling and cell spreading [34]. To examine the effects that IP6K1 has along this pathway, we studied the consequence of its depletion on cell spreading. We held MEFs in suspension in the presence of high (10% serum) or low serum (0.2% serum) growth factors, and replated them on the ECM protein fibronectin, also in high or low serum conditions, allowing them to adhere and spread. Spreading of cells observed 15 min after replating was significantly reduced in *Ip6k1*^−/−^ MEFs, in the presence of both high and low serum (Fig. 2A–D). This suggests that IP6K1 is involved in adhesion-dependent signaling and resulting cytoskeletal remodeling that controls cell spreading, and that IP6K1 acts upstream of integrin-growth factor synergies. All subsequent assays were conducted in the presence of 10% serum, so that cell growth and other growth factor dependent pathways were not compromised.

Reduced cell spreading in *Ip6k1*^−/−^ MEFs could result from defects in integrin engagement with fibronectin. To rule out this possibility, we plated cells on fibronectin and monitored their adhesion to the ECM over different periods of time. Adhesion to fibronectin was comparable in *Ip6k1*^+/+^ and *Ip6k1*^−/−^ MEFs from 5 min to 5 h (Fig. 2E), suggesting that integrin-mediated adhesion is not significantly altered by IP6K1 depletion. Autophosphorylation of focal adhesion kinase (FAK) is triggered by integrin binding to fibronectin and is an early signaling event supporting cytoskeletal remodeling during cell spreading [35], [36], [37]. FAK phosphorylation on Tyr397 was observed in MEFs that were allowed to grow on fibronectin for 4 h (stably adherent cells) (Fig. 2F–G). When MEFs were held in suspension for 90 min, FAK phosphorylation was down-regulated in both *Ip6k1*^+/+^ and *Ip6k1*^−/−^ MEFs, with greater down-regulation seen in *Ip6k1*^−/−^ MEFs (Fig. 2F–G). When cells were subsequently allowed to spread on fibronectin for 20 min, FAK activation was lower in *Ip6k1*^−/−^ compared with *Ip6k1*^+/+^ MEFs (Fig. 2F–G). Paxillin is a scaffold protein that is phosphorylated by FAK and recruits many regulatory and structural proteins required for cytoskeletal reorganization during cell spreading [37], [38], [39]. Phosphorylation changes in Paxillin mirrored those seen in FAK. *Ip6k1*^+/+^ MEFs showed an increase in Paxillin phosphorylation on Tyr118 after plating on fibronectin, whereas the extent of phospho-Paxillin was reduced in *Ip6k1*^−/−^ MEFs (Fig. 2F and H). This indicates that the activation of adhesion-dependent signaling is indeed reduced in the absence of IP6K1. When allowed to spread on fibronectin for 24 h, *Ip6k1*^−/−^ MEFs were still found to marginally lag in their spread area compared with *Ip6k1*^+/+^ MEFs (Fig. 2I–J). However, the two cell types showed no obvious differences in cell morphology and the pattern of F-actin staining by rhodamine-phalloidin (Fig. 2I). These data indicate that IP6K1 is required for early events in integrin-dependent signaling and cytoskeleton remodeling that are involved in cell spreading, while it is dispensable for maintenance of the actin cytoskeleton structure under steady state conditions.

### 3.3 IP6K1 modulates migration in fibroblasts

**Fig. 3:**
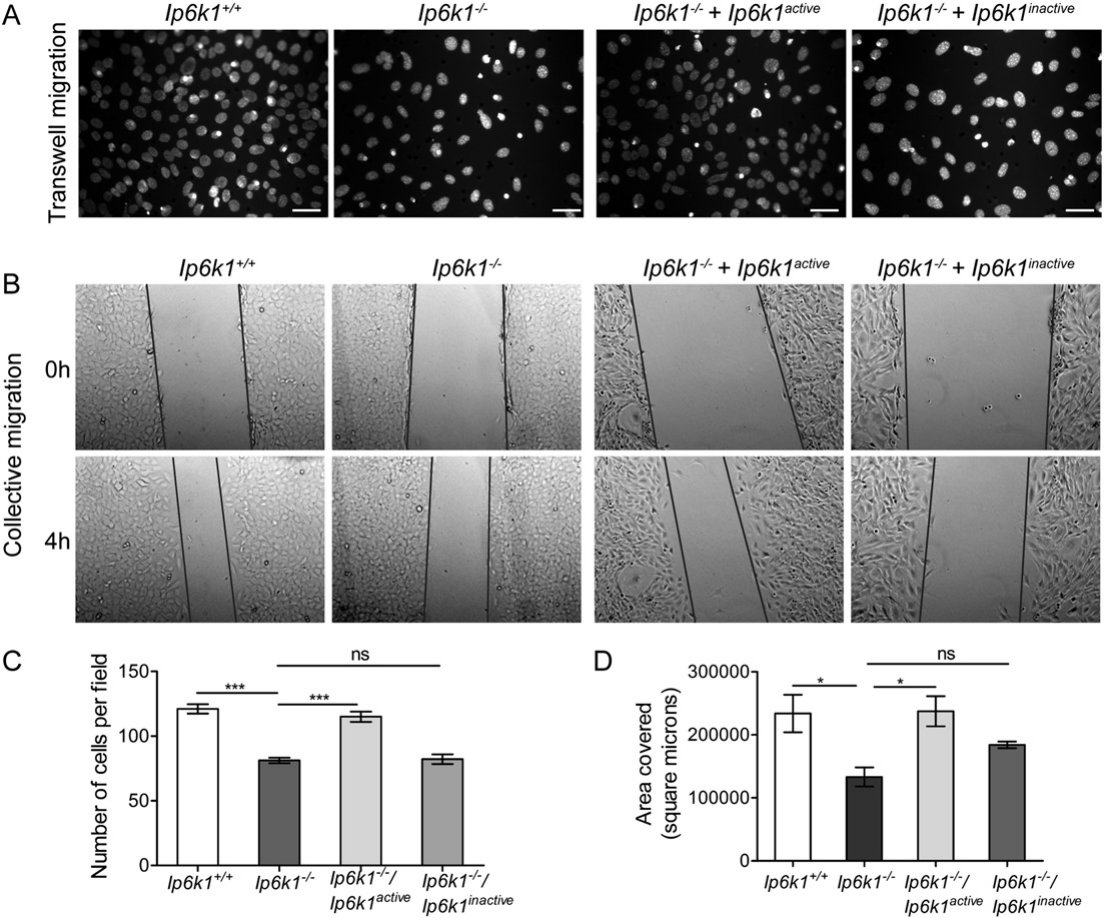
Inositol pyrophosphates modulate cell migration in MEFs. (A) MEFs of the indicated genotypes were seeded in transwell inserts and incubated for 18 h with serum-depleted medium in the upper chamber and complete medium in the lower chamber. Cells that migrated towards serum-rich medium were visualized by staining with DAPI. Scale bars represent 50 μm. (B) Collective cell migration was monitored by a scratch wound healing assay conducted on MEFs of the indicated genotypes. Representative images are shown for the indicated time points. Black lines overlaid on the images mark the edges of the wound. (C) Quantification of (A); the bar graph shows number of cells migrated per field after 18 h. Data represents mean ± SEM (n = 65 and 76 fields respectively for *Ip6k1*^+/+^ and *Ip6k1*^*−*/*−*^ MEFs; n = 77 and 90 fields respectively for *Ip6k1*^*−*/*−*^ MEFs expressing active or inactive forms of IP6K1) compiled from two independent experiments and was analyzed using one-way ANOVA with Tukey's multiple comparison test. (D) Quantification of area covered in (B); data represents mean ± SEM from three independent experiments and was analyzed using one-way ANOVA with Tukey's multiple comparison test. ***P ≤ 0.001; *P ≤ 0.05; ns, not significant, P > 0.05.

Integrin- and growth factor-dependent pathways that regulate cell spreading are also involved in cell migration [33], [34], [37]. This, considered with the down-regulation of genes involved in cell locomotory behavior in *Ip6k*1^−/−^ MEFs (Fig. 1C, Supplemental Table S2), suggests that IP6K1 may influence cell migration and chemotaxis. To test this, we monitored the migration of MEFs plated on permeable membranes in low serum conditions towards serum-rich medium over 18 h. Loss of IP6K1 led to a significant reduction in the number of cells that are able to cross the membrane in response to the growth factor gradient (Fig. 3A and C). We also monitored the collective migration of confluent fibroblast monolayers in an in vitro wound healing assay in the presence of serum growth factors, and noted that wound closure is substantially reduced in *Ip6k1*^−/−^ MEFs (Fig. 3B and D).

The IP6 kinases have been shown to participate in different cellular pathways via the production of inositol pyrophosphates or via interaction with other proteins independent of their enzymatic activity [8]. To determine whether the influence of IP6K1 on chemotaxis and migration is dependent upon its inositol pyrophosphate synthesis activity, we tested *Ip6k1*^−/−^ MEFs expressing either an active or catalytically inactive form of IP6K1 [12]. Active IP6K1 expression was able to restore chemotaxis towards high serum in *Ip6k1*^−/−^ MEFs, whereas the expression of inactive IP6K1 failed to do so (Fig. 3A and C). A similar effect was seen on the defect in collective cell migration, which was completely reversed by expression of active IP6K1, but not by the inactive enzyme (Fig. 3B and D). These data show that IP6K1 can indeed regulate fibroblast migration, and this function is dependent on its ability to synthesize inositol pyrophosphates.

### 3.4 Depletion of IP6K1 impairs migration in cancer cells

**Fig. 4:**
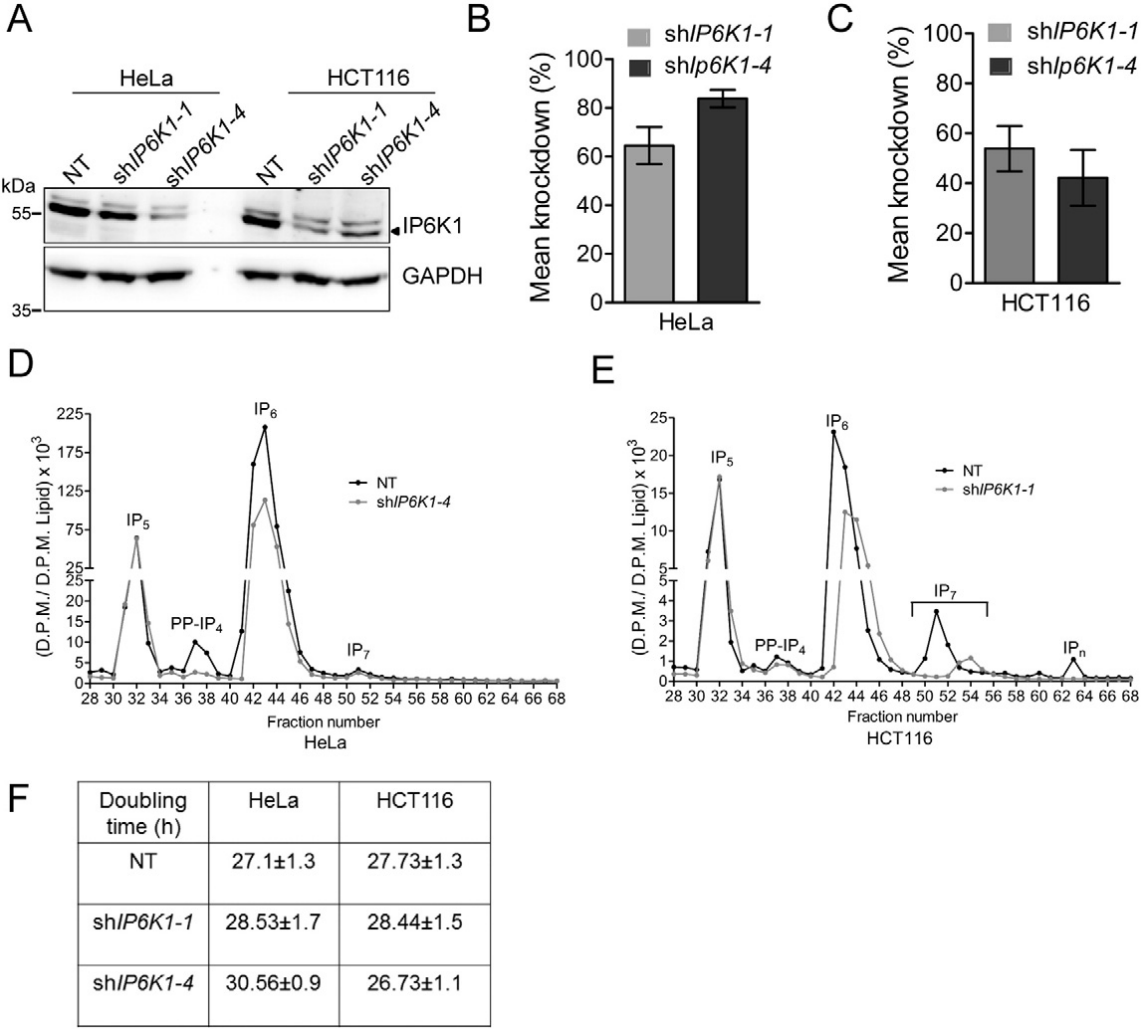
Characterization of cells expressing shRNA against human *IP6K1*. (A) Immunoblot to detect IP6K1 in lysates from HeLa and HCT116 cell lines that stably express the indicated shRNA (NT, non-targeting control; sh*IP6K1*–*1* and sh*IP6K1*–*4*, two different shRNA sequences directed against human *IP6K1*). (B, C) The bar graphs represent IP6K1 knockdown levels in HeLa (B) and HCT116 (C). Data represents mean ± SEM from three independent experiments. (D, E) HPLC profile of [^3^H] inositol labeled HeLa NT and sh*IP6K1–4* (D) and HCT116 NT and sh*IP6K1–1* (E) cell lines. Soluble inositol phosphate counts were normalized to the total lipid inositol count for each sample. Peaks corresponding to IP_5_, PP-IP_4_, IP_6_ and IP_7_ are indicated. Data are representative of two independent experiments. (F) Growth analysis by MTT assay was used to determine the doubling time for HeLa and HCT116 cells stably expressing the indicated shRNA. Data represents mean ± SEM from three independent experiments and was analyzed using one-way ANOVA with Tukey's multiple comparison test. The P value was determined to be > 0.05 across all comparisons, indicating no significant difference in doubling time in cells expressing sh*IP6K1* compared with NT.

To determine whether the effect of IP6K1 on chemotaxis and migration extends to cancer cells, we generated HCT116 and HeLa cell lines expressing shRNA directed against human *IP6K1*. Of the five different shRNA constructs tested, we observed > 30% knockdown in IP6K1 expression with only two constructs, which we called sh*IP6K1*–*1* and sh*IP6K1*–*4*. These shRNA targeted *IP6K1* expression with varying efficiency in HeLa and HCT116 cell lines. While sh*IP6K1*–*4* resulted in an approximately 80% decrease in *IP6K1* expression in HeLa cells compared with control cells expressing non-targeting (NT) shRNA (Fig. 4A–B), the same construct showed poor knockdown efficiency (approximately 40%) in HCT116 cells (Fig. 4A and C). sh*IP6K1*–*1* expression led to an approximately 60% knockdown in both cell lines (Fig. 4A-C). Therefore, sh*IP6K1*–*1* expressing HCT116 cells and sh*IP6K1*–*4* expressing HeLa cells were used for further analysis. Analysis of the soluble inositol polyphosphate profile in HeLa cells expressing sh*IP6K1*–*4* revealed a substantial reduction in the level of the inositol pyrophosphate PP-IP_4_, but no change in the level of its precursor, IP_5_. Interestingly, there was a marginal reduction in IP_6_, but no significant difference in the level of its inositol pyrophosphate product, IP_7_ (Fig. 4D). Conversely, HCT116 cells depleted for IP6K1 showed a significant reduction in IP_7_ and a complete absence of more polar inositol polyphosphates, with a marginal reduction in IP_6_ (Fig. 4E). These cells showed a slight decrease in the level of PP-IP_4_ but no change in IP_5_. Together, our analyses reveal that lowering IP6K1 expression reduces the level of the more abundant inositol pyrophosphate - PP-IP_4_ in HeLa and IP_7_ in HCT116 cells. The concomitant reduction in the level of IP_6_, while unexpected, is supported by the high rate of metabolic turnover reported for inositol pyrophosphates [1], [2], and suggests that cells may evolve compensatory mechanisms in an attempt to maintain the ratio of inositol pyrophosphates to their precursor inositol polyphosphates.

**Fig. 5:**
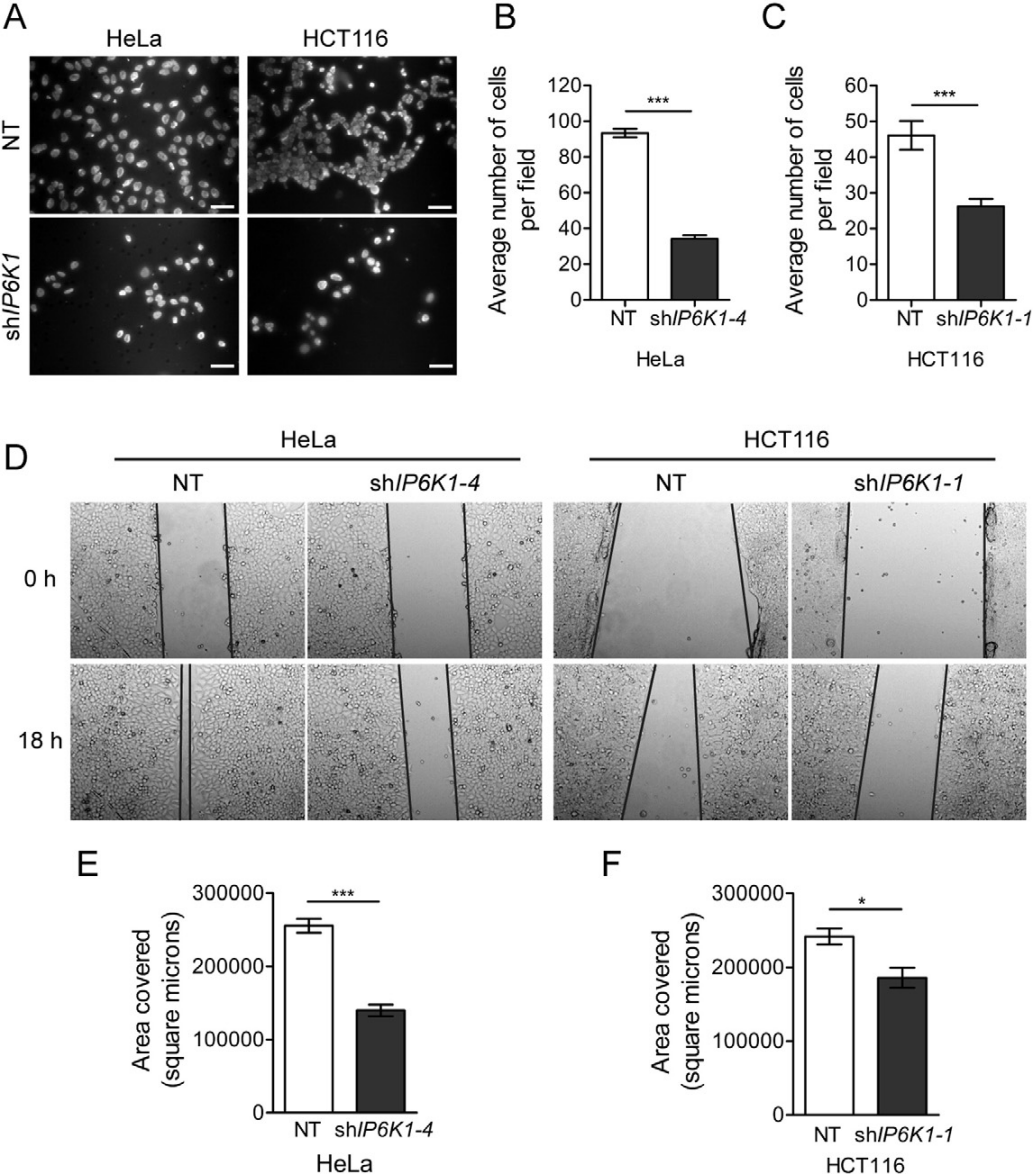
IP6K1 depletion reduces cancer cell migration. (A) Transwell migration was assessed in NT control and sh*IP6K1* expressing HeLa (left panel) and HCT116 cells (right panel). Cells that migrated towards serum-rich medium 24 h after seeding were visualized by staining with DAPI. Scale bars represent 50 μm. (B, C) Quantification of (A); the bar graphs show the average number of cells migrated per field in HeLa (B) or HCT116 (C); data represents mean ± SEM (n = 127 and 134 fields respectively for NT control and sh*IP6K1–4* expressing HeLa; n = 152 and 186 fields respectively for NT control and sh*IP6K1–1* expressing HCT116 cells) compiled from three independent experiments and was analyzed using the non-parametric two-tailed Mann-Whitney test. (D) Scratch wound healing assay on confluent monolayers to monitor collective cell migration in the indicated cell lines. Representative images are shown for the indicated time points. Black lines overlaid on the images mark the edges of the wound. (E, F) Quantification of area covered after 18 h in HeLa (E) or HCT116 (F) cells. Data represents mean ± SEM from three independent experiments and was analyzed using a two-tailed unpaired Student's *t*-test. ***P ≤ 0.001; *P ≤ 0.05.

Depletion of IP6K1 in both HeLa and HCT116 cells resulted in a significant decrease in chemotactic migration towards serum-rich medium over a period of 24 h (Fig. 5A–C). A difference in the rate of proliferation may be reflected as an apparent change in transwell migration. To exclude this possibility we performed a cell proliferation assay and did not observe any significant difference in the doubling time of NT and IP6K1 depleted cells (Fig. 4F). We also performed a wound healing assay on confluent monolayer cultures to look at collective cell migration, and observed reduced migration in IP6K1 depleted HeLa and HCT116 cells (Fig. 5D–F). Together, our data show that the depletion of IP6K1 lowers chemotactic and collective migration in these cancer cell lines.

### 3.5 Depletion of IP6K1 affects the invasive potential of cancer cells

**Fig. 6:**
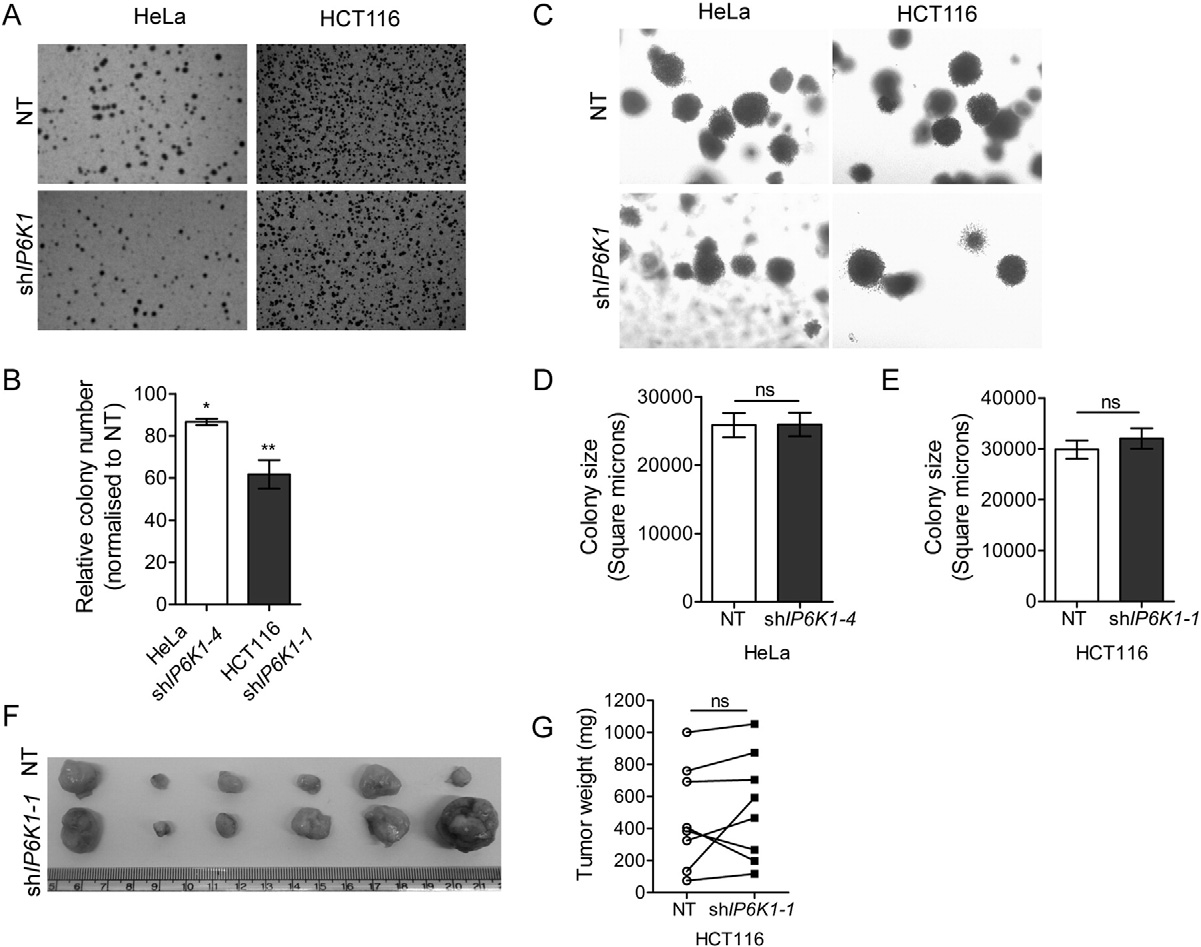
Effect of IP6K1 depletion on tumorigenic potential of cancer cells. (A) Anchorage-independent growth of cells seeded in soft agar was determined after 3 weeks by staining with crystal violet to visualize and count the number of colonies. Representative images of crystal violet stained colonies are shown. (B) Bar graph shows the number of colonies per dish in IP6K1 depleted HeLa and HCT116 cells normalized to their respective NT controls. Data are mean ± SEM from three independent experiments and were analyzed by a one-sample *t*-test. (C) Colonies formed in soft agar (A) were imaged at higher magnification as described in the Methods section. (D, E) Bar graphs showing the colony sizes obtained after growth of HeLa (D) and HCT116 (E) cells for 3 weeks in soft agar (n = 66 and 67 for HeLa NT control and sh*IP6K1–4* respectively; n = 63 and 52 for HCT116 NT control and sh*IP6K1–1* respectively). Data (mean ± SEM) are representative of two independent experiments and were analyzed using the non-parametric two-tailed Mann-Whitney test. (F) Subcutaneous xenografts of HCT116 NT (top row) and sh*IP6K1–1* (bottom row) cells, isolated 4 weeks after injection of cells into either flank of the same mouse. (G) Symbol and line plot displaying the weights of tumors derived from HCT116 NT and sh*IP6K1–1* cells. Symbols indicate the tumor weight scatter, and lines represent the pair-wise analysis of tumors isolated from either flank of the same mouse (n = 8). Data was analyzed using a two-tailed paired Student's *t*-test. **P ≤ 0.01; *P ≤ 0.05; ns, not significant, P ≥ 0.05.

The tumorigenic and metastatic potential of cells depends on their migratory and invasive properties, which require dramatic reorganization of the actin cytoskeleton [40]. Tumorigenicity is further supported by the ability of cells to become anchorage independent via deregulation of integrin-growth factor dependent signaling [41]. We therefore sought to determine the role IP6K1 has on the tumorigenic properties of cancer cells. We conducted soft-agar growth assays to examine anchorage-independent growth of cells expressing *IP6K1*-targeting shRNA. Depletion of IP6K1 led to a decrease in colony number in both HCT116 and HeLa cells (Fig. 6A–B). However, there was no effect of IP6K1 reduction on the size of colonies formed by HeLa or HCT116 cells in soft agar (Fig. 6C–E). We then examined the in vivo tumorigenic potential of HCT116 cells by subcutaneous injections into the flanks of athymic nude mice. Interestingly, we did not observe any significant difference in the size of tumors grown from sh*IP6K1* and NT controls (Fig. 6F–G). This observation correlates with our finding that HCT116 cells expressing sh*IP6K1* show no change in the rate of cell proliferation or colony size in soft agar.

**Fig. 7:**
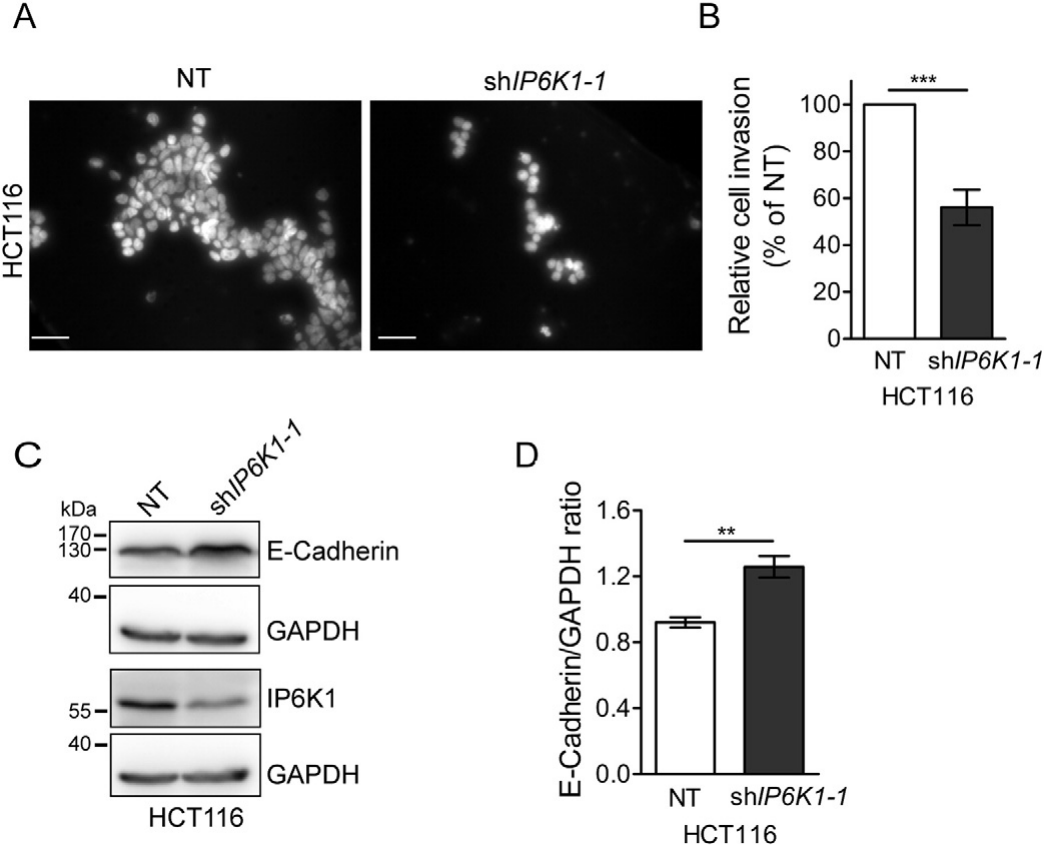
IP6K1 modulates the invasive potential of cancer cells. (A) HCT116 cells stably expressing NT or sh*IP6K1–1* were allowed to invade a matrigel matrix to move towards a high-serum gradient for 24 h. Representative images show cells that migrated through the gel to the other side of the membrane, visualized by staining with DAPI. Scale bars represent 50 μm. (B) Quantification of (A); bar graphs show the number of invaded cells normalized to the NT control. Data are mean ± SEM from five independent experiments, and were analyzed by a one sample *t*-test. (C) Immunoblot analysis of epithelial marker E-Cadherin in HCT116 cells expressing NT or sh*IP6K1–1*. (D) Quantification of (C); levels of the epithelial marker E-cadherin are indicated as a ratio with respect to the levels of GAPDH which was the loading control. Data represents mean ± SEM from three independent experiments and was analyzed using a two tailed unpaired Student's *t*-test. **P ≤ 0.01; ***P ≤ 0.001.

We explored the invasive property of these cells by a matrigel invasion assay, which mimics early steps in tumor metastasis. IP6K1 depleted HCT116 cells showed significantly reduced invasion compared with NT control cells (Fig. 7A–B). Matrigel invasion by epithelial cells has been shown to inversely correlate with the expression of the epithelial biomarker E-cadherin which promotes cell-cell adhesion [25], [42]. We observed higher levels of E-cadherin in sh*IP6K1* cells compared with NT cells (Fig. 7C–D), correlating with their reduced invasion potential. Together, these data suggest that IP6K1 may not play a role in tumor initiation, but is required for aggressiveness of cancer cells by regulating anchorage independence, cell migration and invasion.

### 3.6 Loss of IP6K1 renders mice resistant to 4NQO induced carcinogenesis

**Fig. 8:**
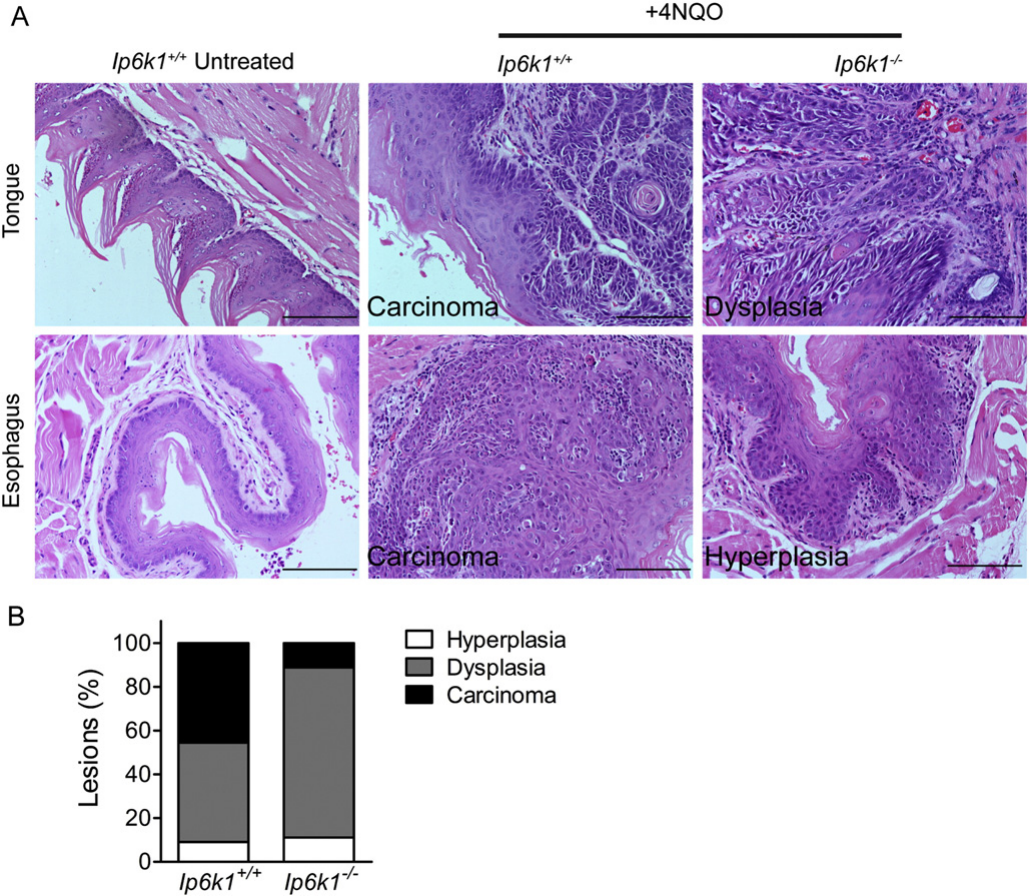
IP6K1 knockout renders mice resistance to 4NQO induced carcinogenesis. *Ip6k1*^+/+^ and *Ip6k1*^*−*/*−*^ mice were administered the oral carcinogen 4NQO in drinking water continuously for 24 weeks. (A) Representative images of H&E stained tissues show the normal epithelium of the tongue and esophagus of untreated *Ip6k1*^+/+^ mice (left panel), induction of invasive carcinoma in the tongue and esophagus of *Ip6k1*^+/+^ mice (middle panel), and the same tissues in *Ip6k1*^*−*/*−*^ revealing dysplasia and hyperplasia (right panel). Scale bars represent 100 μm. (B) Stacked bars represent the percentage of different types of lesions observed in mice of the indicated genotypes. n = 11 and 9 for *Ip6k1*^+/+^ and *Ip6k1*^*−*/*−*^ mice respectively.

To determine if the reduced invasion potential of IP6K1 depleted cancer cells extends to *Ip6k1* knockout cells in vivo we utilized the 4-nitroquinoline 1-oxide (4NQO) oral squamous cell carcinoma model [43]. 4NQO is a water-soluble quinoline derivative, which when administered to mice in drinking water induces a temporal progression of the different phases of carcinogenesis from hyperplasia to dysplasia to invasive carcinoma [24], [44]. 4NQO treatment of cells has been shown to lead to their anchorage independence [45], further supporting a potential role for IP6K1 in 4NQO induced carcinogenesis. After 24 weeks of continuous exposure to 4NQO, we observed 100% survival in both *Ip6k1*^+/+^ and *Ip6k1*^−/−^ mice. Histopathological examination of tissues from the upper aerodigestive tract revealed hyperplasia and dysplasia in tongue and esophagus of both *Ip6k1*^+/+^ and *Ip6k1*^−/−^ mice (Fig. 8A–B). However, invasive carcinoma, defined by the migration of dysplastic epithelial cells into subepithelial tissues, was less in case of *Ip6k1*^−/−^ mice, suggesting that these mice are protected against 4NQO induced carcinogenesis. Taken together, our studies in cells and mice lacking IP6K1 identify a role for this protein in coordinating multiple cellular events to regulate cell migration, invasion and carcinogenesis.

## 4 Discussion

Our study reveals that IP6K1 facilitates the migration and invasion of cancer cells, so that mice lacking IP6K1 show reduced progression from epithelial dysplasia to invasive carcinoma upon chronic exposure to an oral carcinogen. At the molecular level, the absence of IP6K1 brings about changes in gene expression resulting in the down-regulation of pathways involved in signaling between the cell surface and the extracellular matrix. This leads to deficiencies in integrin-mediated signaling events such as FAK and Paxillin phosphorylation, and defects in actin cytoskeleton remodeling, resulting in reduced spreading, motility, anchorage-independent growth and invasion.

The migration defects we observed in fibroblasts lacking IP6K1 were completely restored upon expression of active but not inactive IP6K1, suggesting that inositol pyrophosphate synthesis is required to support cell migration. Migration and invasion are complex cellular responses that receive inputs from multiple signaling pathways including extracellular matrix-cell surface signaling, growth factor signaling, actin cytoskeleton remodeling, and the secretion of matrix proteases. IP6K1 may impinge upon some or all of these pathways directly, or act indirectly by altering gene expression. IPMK, a related member of the inositol phosphate kinase family has been shown to regulate mRNA transcription and export via the synthesis of nuclear PI(3,4,5)P_3_, influence epigenetic regulation via its product I(1,4,5,6)P_4_, and also act directly as a transcriptional co-activator in an enzyme-independent manner [46]. Similarly, IP6K1 could regulate gene expression via the synthesis of inositol pyrophosphates or by directly interacting with other proteins. Inositol pyrophosphates have been shown to control the activity of the histone deacetylase Rpd3L in yeast [47] and the histone demethylase JMJD2C in mammals [48] to regulate chromatin remodeling. The inositol pyrophosphate 1-IP_7_ has been shown to activate the mammalian transcription factor IRF3 [49]. Inositol pyrophosphates synthesized by IP6K1 could also act in a similar manner, influencing the activity of transcription regulatory proteins via serine pyrophosphorylation or binding.

Inositol pyrophosphates synthesized by IP6K1 have been shown to play an important role in maintaining genomic integrity by promoting DNA repair through homologous recombination [12] and nucleotide excision repair pathways [13], suggesting that mice lacking *Ip6k1* may exhibit a propensity to develop spontaneous tumors. However, gross and microscopic examination of liver, kidney, spleen, thymus and lymph nodes from four individual 2 year old male *Ip6k1*^+/+^ and *Ip6k1*^−/−^ mice revealed no lesions, suggesting that the loss of IP6K1 does not result in spontaneous tumor formation. Despite their overlapping expression pattern and high degree of sequence identity, IP6K1 and IP6K2 have several distinct cellular and physiological functions. IP6K1 promotes insulin secretion from pancreatic beta cells [11] and mice lacking IP6K1 display reduced levels of serum insulin [14], whereas IP6K2 knockout mice have normal insulin levels [21]. Male mice lacking IP6K1 are sterile [14] whereas IP6K2 knockout mice show normal fertility [21]. At the cellular level, MEFs derived from *Ip6k1*^−/−^ mice displayed a 70% reduction in IP_7_ levels [14] whereas *Ip6k2*^*−*/*−*^ MEFs showed a 20 to 30% reduction [20], [21], suggesting that IP6K1 is the major IP_7_ synthesizing enzyme in these cells. HCT116 cells have been shown to possess very high levels of inositol pyrophosphates [50]. The deletion of *IP6K2* in these cells leads to an almost complete loss of IP_7_ [19], whereas we observe that depletion of IP6K1 also results in a decrease in IP_7_ levels (Fig. 4E), suggesting that both IP6K1 and IP6K2 contribute to IP_7_ pools in HCT116 cells.

Recently, it was shown that HCT116 cells lacking IP6K2 display defects in cell adhesion and migration, resulting in reduced tumor growth and metastasis when these cells are implanted in immunocompromised mice [18]. Complementation with the active but not the inactive enzyme was able to reverse defects in cell migration and tumor growth, suggesting that inositol pyrophosphates are essential for these processes. Although we also observe reduced migration and invasion properties in IP6K1 depleted HCT116 cells, there is no reduction in tumor volume. We speculate that the threshold level of inositol pyrophosphates required to support cell migration is higher than the level required for tumor growth. Therefore, the depletion of PP-IP_4_ and IP_7_ in sh*IP6K1–1* HCT116 cells is sufficient to result in migration and invasion defects but not enough to cause a decrease in tumor volume, whereas the near complete loss of IP_7_ in *IP6K2* knockout HCT116 cells affects both these processes [18]. The distinct functions of IP6K1 and IP6K2 in tumor development may also arise from their differential effects on cell proliferation, with *IP6K2* knockout HCT116 cells exhibiting delayed growth [18], and IP6K1 depleted cells exhibiting no change in doubling time (Fig. 4F).

Although HCT116 cells lacking IP6K2 show reduced tumor development [18], mice lacking IP6K2 show accelerated development of invasive aerodigestive tract epithelial carcinoma upon being fed the oral carcinogen 4NQO [21]. This was attributed primarily to the apoptosis-enhancing functions of IP6K2 as well as to overexpression of oncogenes and loss of tumor suppressors in *Ip6k2* knockout tissues. Our results upon feeding the same carcinogen to *Ip6k1* knockout mice revealed that unlike IP6K2, the loss of IP6K1 protects them from invasive carcinoma, underlining the fact that IP6K1 and IP6K2 have distinct roles in carcinogenesis. One possible reason for this divergence between the functions of IP6K1 and IP6K2 is that the loss of IP6K2 but not IP6K1 has been shown to render cells resistant to apoptosis [51]. Another reason could be distinct patterns of gene expression in *Ip6k1* versus *Ip6k2* knockout mice. The alterations in expression of oncogenes and tumor suppressors identified in *Ip6k2* knockout tissues [21] may not be conserved in *Ip6k1* knockout mice. Taken together, these studies reveal that in aerodigestive tract epithelial cells it is IP6K1 that is responsible for carcinogenesis, whereas the dominant function of IP6K2 is to protect cells from transformation. Therefore, the development of an isoform-specific IP6K1 inhibitor may provide a novel route to prevent the progression of epithelial dysplasia to invasive carcinoma.

## 5 Conclusions

In the present study we demonstrate the importance of IP6K1 in adhesion-dependent signaling pathways controlling cell spreading and migration. Cancer cells deficient in IP6K1 show decreased anchorage independent growth, migration and invasion. Mice lacking IP6K1 are protected from invasive carcinoma induced by an oral carcinogen, suggesting that IP6K1 inhibition may provide a novel path to suppress cancer progression.

The following are the supplementary data related to this article.

Supplemental Table S1 List of primers used in RT-qPCR analysis. Supplemental Table S1

Supplemental Table S2 List of up- and down-regulated genes and Functional Annotation Clustering of up- and down-regulated genes in *Ip6k1*^−/−^ MEFs when compared with *Ip6k1*^+/+^ MEFs. Supplemental Table S2

## Author contributions

RSJ conducted most of the experiments, analyzed the results, and wrote most of the paper. DK and NBu conducted experiments on cell adhesion and spreading. SG performed RT-qPCR analysis and E-caherin Western blot analysis. SRT performed inositol polyphosphate determination experiments. NBa provided expertise on cell surface-ECM signaling and cell spreading. RB conceived and coordinated the study and wrote the paper with NBa and RSJ. All the authors read and approved the final version of the manuscript.

## Funding information

This work was supported by a Senior Fellowship from the Wellcome Trust/Department of Biotechnology India Alliance (WT/DBT IA, 500020/Z/09/Z to R.B.) and Centre for DNA Fingerprinting and Diagnostics core funds. R.S.J. and S.R.T. are recipients of Junior and Senior Research Fellowships from the Council of Scientific and Industrial Research and the Department of Biotechnology, Government of India, respectively. N·Ba acknowledges funding from a WT/DBT IA Senior Fellowship (WT_DBT_30711059).

## Conflict of interest

The authors declare that they have no conflicts of interest with the contents of this article.
