# Supplementary material for: Deletion of inositol hexakisphosphate kinase 1 (IP6K1) reduces cell migration and invasion, conferring protection from aerodigestive tract carcinoma in mice
Source: Cell Signal. 2016 Aug;28(8):1124–36. doi: 10.1016/j.cellsig.2016.04.011 (PMC4913618; doi:10.1016/j.cellsig.2016.04.011)
Supplement: Supplemental Table S1 — List of primers used in RT-qPCR analysis. [file mmc1.pdf]

**Supplementary Table S1**

List of primers used for RT-qPCR

| Gene | RT-qPCR primers |
|---|---|
| *Ifi202b* | F: 5'-AAAGTATACAGGAAAAGTACCGGA- 3'<br>R: 5'-TCTCAGAAACCACTCATCTGCAT- 3' |
| *Zfp560* | F: 5'-AGAGTTCACCAAGACTGGGC- 3'<br>R: 5'-ACTGCATGGAGGCTCTTTGA- 3' |
| *Pdgfb* | F: 5'-CAAGACGCCTCAAGCTCGG- 3'<br>R: 5'-AAATAACCCTGCCCACACTCTT- 3' |
| *Iqgap2* | F: 5'-GGCAGCAAAGCTACATGACA- 3'<br>R: 5'-CCACCCCTTCATACTGCATC- 3' |
| *Coro2a* | F: 5'-CCAGTGCTTCCCATCAAA -3'<br>R: 5'-CCCAACAGAACAAGAAACAACA -3' |
| *Tm6sf1* | F: 5'-AGAGGCTTGATTGCTTTGGA- 3'<br>R: 5'-GAGCATCCAGGGACCACTAA-3' |
| *Slc15a2* | F: 5'-TATCACCAACCGGGGTCTTC- 3'<br>R: 5'-CATGCTAGATGGTGCCTGAGA- 3' |
| *Arg1* | F: 5'-ACAAGACAGGGCTCCTTTCAG- 3'<br>R: 5'-GGCTTATGGTTACCCTCCCG- 3' |
| *Dmbt1* | F: 5'-TCCACAGTCAGCACAAGTCC- 3'<br>R: 5'-CAGCTGTCATCACACACGGT- 3' |
| *Paip1* | F: 5'-CATCTGATGGTGTTCCTTTCACT-3'<br>R: 5'-CGATATCATCCAAATATGGGTCAC-3' |
| *Edn1* | F: 5'-TGCTGGAATTTCTGCCAAGC- 3'<br>R: 5'-TGGCCTCCAACCTTCGTAGT- 3' |
| *Hoxc13* | F: 5'-TCCCTTCCCAGACGTGGTTC-3'<br>R: 5'-CGGCGCTTCTCTTTGGTGAT-3' |
| *Nup210* | F: 5'-CCTGTCACACTTCCTGGACTC- 3'<br>R: 5'-GAGGCAAGATAGTGGGGGCT- 3' |
| *Sdcbp2* | F: 5'-TGGGCTAAAGGACAAAAAGG-3'<br>R: 5'-AGGTGTCTGGAATGGAGTGG-3' |
| *Ddx27* | F: 5'-GGATCCTTCCCCAGGATGTTA- 3'<br>R: 5'-GTCGCTGTGCTGTGTCAATC- 3' |
| *Dscam* | F: 5'-CGGCCAGGCGACCTCATAC- 3'<br>R: 5'-GGTCCGACTTTTCTGGGGTT- 3' |
| *Stx3* | F: 5'-TGCTGGTGGAAAATCAGGGAG- 3'<br>R: 5'-AAGCCAAGATGATGGCAAGGA- 3' |
| *Prkg2* | F: 5'-TATGAGCTCCTAACGGGCAAC- 3'<br>R: 5'-CTTTCTGTTGGGTTTTGCCTGC- 3' |
| *Taf9* | F: 5'-GGCCTTTGTCAATCTGGTACAC- 3'<br>R: 5'-TCTTGGGAGACGCCATCTTG- 3' |
| *Fgd3* | F: 5'-TGATGCTCTACTGTGTACCCAAA-3'<br>R: 5'-GCTCCAGGGACCTTTTTCTT-3' |
| *Ctsw* | F: 5'-TCCATTCCAGGGGGACAGAA- 3'<br>R: 5'-TCTGGTAATGCTGGAGTAGTTTCA- 3' |
| *Cpn1* | F: 5'-CCGGGAAGCCTTAATCCAGTT- 3'<br>R: 5'-AATAATCCCCATGTTCACCTGAAGT- 3' |

| | |
|---|---|
| *Ifna12* | F: 5’-TGCTGGCAAGACTGAGTGAG- 3’<br>R: 5’-ACTGTTGAAACATCAAGGCAACT- 3’ |
| *Rbp1* | F: 5’-GCCTTACGCAAAATCGCCAA-3’<br>R: 5’-CAGTGGTCATGCACTTGCG-3’ |
| *Pon1* | F: 5’-GGTCCTCAACTTTGACACCCT-3’<br>R: 5’-ATTCGAAGCACCTCTGAGCC-3’ |
| *Cxcl5* | F: 5’-TCAGAAAATATTGGGCAGTGACAA-3’<br>R: 5’-GAAAATCCGTGGGTGGAGAGA-3’ |
| *Smoc1* | F: 5’-CGATGACATTAACAAGCGGGAG-3’<br>R: 5’-CTACCACCTTCTTTGCTAACACC-3’ |
| *Mgp* | F: 5’-GTGCTACGAATCTCACGAAAGC-3’<br>R: 5’-TGTTGATCTCGTAGGCAGGC-3’ |
| *Selenbp1* | F: 5’-ATGCCTGGTTTGATCACCGA-3’<br>R: 5’-CTCTAACAATGCTGCCCCCA-3’ |
| *Meox1* | F: 5’-AAAGAGAGGTCAGACAACCAGGA-3’<br>R: 5’-GAGCCGGGTCAGGTAGTTGT-3’ |
| *Slfn2* | F: 5’-GGCTGCAGTTAGAACTCTTCCA-3’<br>R: 5’-GTCATTCCTCTGAGTTCTGTGGT-3’ |
| *Palmd* | F: 5’-AACTGCAAATCTCAGCCAACG-3’<br>R: 5’-TGTCCTCGATTGACTCTTCTGG-3’ |
| *Rarres2* | F: 5’-AATTCTGAAGCAAGGGCCTC-3’<br>R: 5’-GGGAGTAAGGTGAAGTCCTGTC-3’ |
| *Ptprz1* | F: 5’-TCTACACCACCAACACCCATC-3’<br>R: 5’-AGCTCTGCACTTCCTGGTAA-3’ |
| *Podn* | F: 5’-GGACATCGCTGGGAATCAGC-3’<br>R: 5’-AGCCAGCTTGTTGAACCTGAG-3’ |
| *Arsk* | F: 5’- ATCTACCCTACTATGCTTGACATTG-3’<br>R: 5’- TGCATCCATGGAATTCACTC-3’ |
| *Aldh3b1* | F: 5’-CCCCCACAGTGTTAGTGGAC-3’<br>R: 5’-TGATAACCTGGCTTCTCTTGGA-3’ |
| *Ccl17* | F: 5’-ATGCCATCGTGTTTCTGACT-3’<br>R: 5’-GGTCACAGGCCGTTTTATGT-3’ |
| *Pcdhga1* | F: 5’-CACTCACTGCTGACTCTCGAA-3’<br>R: 5’-GCGGGGCTTGCTGAGAAAATA-3’ |
| *Espn* | F: 5’-GGCCATATACCCAGCACCAA-3’<br>R: 5’-ACTGCCTGAGAACACGGTTG-3’ |
| *Cxcl15* | F: 5’-CTGCTGGCTGTCCTTAACCT-3’<br>R: 5’-GGGACTGCTATCACTTCCTTTCT-3’ |
| *Fgf21* | F: 5’-CTGGGGGTCTACCAAGCATA-3’<br>R: 5’-CACCCAGGATTTGAATGACC-3’ |
| *Mmp9* | F: 5’-CAAGGACGGTTGGTACTGGAA-3’<br>R: 5’-AAAACCCTCTTGGTCTGCGG-3’ |
| *Mmp19* | F: 5’-GATCCAGGCTCTCTATGGCAAG-3’<br>R: 5’-AGTCGCCCTTGAAAGCATAAGT-3’ |
| *Itga8* | F: 5’-ACACGTTCCTCAAGAGAAAGAATG-3’<br>R: 5’-ACATTCGGAGTGGCCCAAAT-3’ |
| *Mylk* | F: 5’-GGCAGGAAATCTTCAACAGGG-3’<br>R: 5’-TCCACGACTTCCAGATCACG-3’ |
| *Igfbp3* | F: 5’-AAGAAGAAGCAGTGCCGCC-3’<br>R: 5’-ATGTGGCACGGAGCATCTAC-3’ |

| | |
|---|---|
| *Triobp* | F: 5’-CAATTTCTTGGTGGCGTGT-3’<br>R: 5’-GTTGACTCACCCTGGCTCTC-3’ |
| *Gapdh* | F: 5’- GGAGAAGGCCGGGGCCCACTTGAA-3’<br>R: 5’- GCATGGACTGTGGTCATGAGCCCTTCCAC-3’ |
